# The prolonged effect of glucagon-like peptide 2 pretreatment on growth performance and intestinal development of weaned piglets

**DOI:** 10.1186/s40104-016-0087-7

**Published:** 2016-05-04

**Authors:** Qiu Hong Deng, Gang Jia, Hua Zhao, Zheng Li Chen, Xiao Ling Chen, Guang Mang Liu, Kang Ning Wang

**Affiliations:** Animal Nutrition Institute, Sichuan Agricultural University, Chengdu, Sichuan 611130 China; College of Animal Medicine, Sichuan Agricultural University, Chengdu, Sichuan 611130 China

**Keywords:** *Escherichia coli* lipopolysaccharide, Glucagon-like peptide-2, Growth performance, Intestinal enzymes, Small intestinal morphology, Weaned piglets

## Abstract

**Background:**

Glucagon-like peptide 2 (GLP-2) is a potent epithelium-specific intestinal growth factor. The aim of this study was to demonstrate the prolonged effect of GLP-2 on the growth performance of weaned piglets. Forty piglets weaned at the age of 28 d with an average BW of 6.8 ± 0.4 kg were assigned to four treatments: (i) non-challenged control; (ii) LPS-challenged control; (iii) LPS + low GLP-2; and (iv) LPS + high GLP-2. Piglets in groups (i), (ii), and (iv) were s.c. injected with PBS supplemented with human [Gly2]GLP-2_1-34_ at doses of 0, 2 and 10 nmol/kg BW per day for seven consecutive days. BW, gain:feed ratio (G:F), and plasma GLP-2 levels were determined on d 0, 7, and 14 after weaning. Piglets were challenged with i.p. administration of *Escherichia coli* lipopolysaccharide (LPS) at a dose of 100 μg/kg on d 14 to induce intestinal damage. Twenty-four hours later, intestinal tract samples were collected to assess intestinal morphology and quantify enzyme activity.

**Results:**

Plasma GLP-2 levels decreased after weaning, but in the high GLP-2 group, plasma GLP-2 was maintained on d 7 and even increased to a level higher than the preweaning level on d 14 (*P <* 0.05). High GLP-2 treatment significantly increased the duodenal, jejunal and ileal weight, as well as the gross weight of the small intestine (SI), and the SI weight index (*P <* 0.05). LPS caused villous atrophy and disrupted intestinal morphology in the duodenum, jejunum and ileum. GLP-2 also significantly increased the villus height and the villus height/crypt depth ratio (VCR) of the duodenum, jejunum, and ileum (*P* < 0.05). Histological examination revealed that in GLP-2-treated groups, the integrity of the villus was maintained, and the villus was protected against LPS-induced damage. GLP-2 significantly increased the activity of alkaline phosphatase (AKP), γ-glutamyltranspeptidase (γ-GT), and pancreatic lipase in the duodenum and jejunum (*P <* 0.05). GLP-2 treatment also significantly increased the average daily gain (ADG) and G:F of piglets at 0 to 7, 7 to 14, as well as 0 to14 d (*P <* 0.05), resulting in a significant increase of final BW in high GLP-2 pigs (*P =* 0.016).

**Conclusions:**

Exogenous GLP-2 improved the growth of weaned piglets and protected them against LPS-induced intestinal damage. These effects may be due to the ability of GLP-2 to promote the secretion of endogenous GLP-2 to stimulate the small intestinal development.

## Background

Postweaning intestinal disorders, characterized by decreased intestinal enzyme activity and abnormal intestinal morphology, cause serious economic losses in the swine industry. Digestive enzymes such as trypsin, amylase, and lipase are reported to exhibit minimum activity at 5 d postweaning, resulting in poor digestibility [1]. Prevention of intestinal villous atrophy can improve the growth performance of weaned piglets by enhancing nutrient digestion and absorption [2]. Glucagon-like peptide 2 (GLP-2), a 33-amino-acid proglucagon-derived peptide, is a specific intestinotrophic hormone secreted from enteroendocrine L cells. It has been shown to improve intestinal structure development in fetal and neonatal piglets [3, 4] and also to play a regulatory role in intestinal adaptation in obese mice [5]. Plasma GLP-2 concentration was reported to decrease with weaning-related anorexia and increase with resumption of feed intake [6], indicating that GLP-2 may play a role in the growth of the small intestine during weaning via a response to enteral nutrition. Considering that GLP-2 can enhance the proliferation and differentiation of epithelial cells [7], we hypothesize that exogenous GLP-2 can improve the growth performance of weaned piglets and protect their intestinal mucosa from stress damage after weaning by increasing the secretion of endogenous GLP-2 and promoting intestinal development. Currently there is limited research on the effects of GLP-2 on the endogenous GLP-2 secretion and growth in weaned piglets. In the present study, *Escherichia coli* lipopolysaccharide (LPS) was injected into piglets to establish an intestinal injury model, as was previously reported by Liu et al. [8]. The aim of this study was to investigate the prolonged effects of exogenous GLP-2 on growth, intestinal morphology, and enzyme activity in weaned piglets challenged with LPS.

## Methods

### Animal care and diets

Animal care protocols and experimental procedures in this study were approved by the Animal Care and Use Committee of Sichuan Agricultural University. Forty castrated male crossbred piglets (Duroc × Landrace × Yorkshire) with average initial BW of 6.8 ± 0.4 kg were selected from eight litters and weaned at 28-day of age. The piglets had access to a commercial cereal-based diet (Cargill, China) from 21-day of age. Animals were housed individually with free access to water and feed. They were fed four times daily at approximately 0700 h, 1100 h, 1500 h, and 1900 h, with surplus feed (10~15 g) left in the trough feeder each time. The animals were housed in an environmentally regulated nursery maintained at a temperature of 26 °C.

### Experimental design

At 28-day of age (the weaning day), piglets were assigned to four treatments: (i) non-challenged control (control; piglets injected with sterile saline on d 14 postweaning); (ii) LPS-challenged control (LPS; piglets injected with sterile PBS solution containing 0 nmol/kg BW human GLP-2 analog (h[Gly2]GLP-2_1-34_) during the first 7 d postweaning, and then challenged by injection with LPS on d 14 postweaning); (iii and iv) low and high GLP-2 groups (piglets injected with PBS solution containing h[Gly2]GLP-2_1-34_ at doses of 2 and 10 nmol/kg BW per day for the first 7-day after weaning [3], and then challenged by injection with LPS on d 14 postweaning). The PBS solutions containing h[Gly2]GLP-2_1-34_ (Phoenix Pharmaceuticals, Inc., USA, purity ≥ 95 % [HPLC]) for the three treatments (excluding control) were prepared based on an initial BW of 6.8 kg with an injection vol of 1 mL. These solutions were s.c. injected at the nape twice daily at 0800 h and 2000 h. Each experimental group comprised ten piglets, with one piglet per pen, treated over a 15-day period. In this study, d 0 was the weaning day. On d 14 postweaning, the piglets were treated with an i.p. injection of LPS (100 μg/kg BW, Sigma, USA) dissolved in sterile saline to establish an intestinal injury model. According to the previous study by Zhu et al. [9], to avoid the possible effects of LPS-induced feed intake reduction on intestinal characteristics, all piglets in the four treatment groups received 20 g feed/kg BW during 14–15 d (post-challenge). BW and feed intake were determined on d 7 and 14 postweaning to evaluate the average daily gain (ADG), average daily feed intake (ADFI), and gain:feed ratio (G:F).

### Blood and intestinal sample collection

**Fig. 1:**
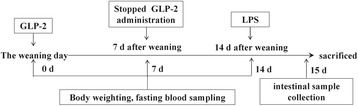
The time flow chart for treatment and sample collection

The time flow chart for treatment and sample collection is shown in Fig. 1. On d 0, 7, and 14, fasting blood was collected from the vena cava after an overnight fast for determination of GLP-2 concentration. Blood samples were kept in precooled vials containing EDTA (2 mg per mL of blood) and aprotinin (400 kIU per mL of blood), and were gently rocked several times to inhibit the activity of proteinases. The blood was centrifuged at 1500 × *g* for 15 min at 4 °C to collect the plasma. Plasma samples were then stored at -20 °C. Plasma GLP-2 concentrations were determined using a commercial RIA kit (Phoenix Pharmaceuticals, Inc., USA). This assay recognizes the synthetic form of the human and porcine GLP-2 peptides.

Twenty-four hours after the administration of LPS (on d 15), all pigs were sacrificed by sodium pentobarbital anesthesia (50 mg/kg BW, i.v.). The abdomen was quickly opened, and the gastro-intestinal tract was dissected and cleaned. Each segment of small intestine was then gently blotted dry and weighed. After digesta content was collected, 20-cm segments were excised from both the mid-duodenum and mid-jejunum to collect mucosal samples by glass slide scraping. Digesta content and mucosal samples were placed in cryovials and frozen in liquid nitrogen for enzyme activity assays. A 4-cm segment was obtained from the mid-duodenum, mid-ileum, and mid-jejunum for subsequent histological analysis. These samples were rinsed with 0.1 mol/L PBS at pH 7.2, and then fixed with 4 % polyformaldehyde.

### Enzyme activity assay

Enzyme extracts were prepared from intestinal mucosa using a procedure modified from Moffa et al. [10]. The mucosa was manually homogenized with a cold glass/glass homogenizer and then disrupted by an ultrasonic cell-break method in 10 vol (wt/vol) of ice-cold sodium chloride solution (0.1 mol/L). The homogenates and digesta were centrifuged at 20,000 × g for 30 min at 4 °C, and the supernatants were collected for determination of protein content and enzyme activity. The enzyme activity level for each sample was normalized against the corresponding total protein content and was expressed as units per gram or milligram of protein. The intestinal digesta and mucosal protein concentrations were determined using a Coomassie (Bradford) protein assay kit (Thermo Scientific Pierce Protein Research Products, USA). Bovine serum albumin was used as the protein standard. Enzyme activity was determined using commercial colorimetric assay kits (Nanjing Jiancheng Bioengineering Institute, China). One unit of alkaline phosphatase (AKP) activity was defined as the activity liberating 1 mg phenol in 15 min at 37 °C per gram of intestinal mucosal protein. One unit of Na^+^-K^+^-ATPase was defined as the amount of ATPase that hydrolyzes ATP to yield 1 μmol inorganic phosphorus per hour per milligram of mucosal protein at 37 °C. One unit of γ-glutamyltranspeptidase (γ-GT) activity was defined as the activity liberating 1 μmol of paranitroaniline in 10 min at 37 °C per milligram of intestinal mucosal protein. One unit of amylase activity was defined as the activity hydrolyzing 10 mg starch in 30 min at 37 °C per milligram of intestinal digesta protein. One unit of lipase activity was defined as the activity hydrolyzing 1 μmol triacylglycerol in 1 min at 37 °C per gram of intestinal digesta protein.

### Morphological analysis

Paraffin sections (4 μm thick) were prepared from polyformaldehyde-fixed samples of the duodenum, jejunum, and ileum, and were stained with hematoxylin and eosin. The mucosal structure was observed using a Nikon 50i-BF fluorescent biomicroscope (Nikon Co., Japan), and feature dimensions were measured using Image-Pro Plus (IPP) image analysis software. At a magnification of 100 ×, the villus height and crypt depth of ten well-oriented villi were measured. Villus height was measured from the crypt opening to the tip of the villus, while crypt depth was measured from the base of the crypt to the level of the opening [11]. Values are expressed as means from ten adjacent villi. The villus height/crypt depth ratio (VCR) was calculated.

### Statistical analysis

Data for the four treatment groups was analyzed using SPSS 17.0 statistical software. Data was first analyzed by one-way ANOVA with GLP-2 injection dose as a main effect, followed by a LSD means comparison test to compare the differences among treatments. The difference was considered significant if *P <* 0.05.

## Results

### Growth performance

BW, ADFI, ADG, and G:F (pre-LPS challenge) are shown in Table 1. The low GLP-2 treatment resulted in a trend of increased final BW (*P =* 0.061), whereas high GLP-2 significantly increased the final BW compared with the pre-LPS pigs (*P =* 0.022). No significant difference in ADFI was observed regardless of the treatment. However, both low and high GLP-2 treatments significantly increased ADG at 0 to 7 (*P =* 0.013 and 0.019), 7 to 14 (*P =* 0.052 and 0.004), and 0 to 14 d (*P =* 0.018 and 0.003). After the first 7-day period, low and high doses of GLP-2 significantly increased the G:F by 18.78 % and 20.81 %, respectively, compared to the pre-LPS group (*P <* 0.001 and *P <* 0.001). After the last 7-day of the experiment, low and high doses of GLP-2 increased the G:F by 16.54 % and 26.47 %, respectively (*P =* 0.005 and *P <* 0.001). Overall, during the 14-day experimental period, low and high doses of GLP-2 increased the G:F by 17.43 % and 23.67 %, respectively (*P =* 0.001 and *P <* 0.001).

**Table 1:** The effect of h[Gly2]GLP-2_1-34_ on the growth of weaned piglets before LPS challenge^a^

Item	Treatment^b^				Pooled SEM	Contrast^c^		
	Control	LPS	LPS + Low GLP-2	LPS + High GLP-2		1	2	3
Initial BW, kg	6.78	6.82	6.82	6.78	0.068	0.824	0.980	0.844
Final BW, kg	9.61	9.41	9.98	10.11	0.11	0.499	0.061	0.022
d 0 to 7								
ADFI, g/d	320	321	320	312	7	0.966	0.943	0.691
ADG, g/d	183	174	205	203	5	0.465	0.013	0.019
G:F	0.574	0.543	0.645	0.656	0.012	0.237	<0.001	<0.001
d 7 to 14								
ADFI, g/d	379	355	380	394	9	0.360	0.340	0.148
ADG, g/d	221	195	245	272	10	0.306	0.052	0.004
G:F	0.583	0.544	0.634	0.688	0.013	0.198	0.005	<0.001
d 0 to 14								
ADFI, g/d	350	338	350	353	7	0.558	0.552	0.468
ADG, g/d	202	184	225	237	7	0.297	0.018	0.003
G:F	0.579	0.545	0.640	0.674	0.012	0.196	0.001	<0.001

^a^GLP-2 = Glucagon-like peptide 2; LPS = *Escherichia coli* lipopolysaccharide

^b^Control: non-challenged control; LPS: pigs injected with sterile PBS solution containing 0 nmol/kg BW human GLP-2; LPS + Low and LPS + High GLP-2: piglets injected with PBS solution containing h[Gly2]GLP-2_1-34_ at doses of 2 and 10 nmol/kg BW per day for the first 7 d after weaning. The data were obtained before LPS challenge

^c^Contrast: (1) control v. LPS; (2) LPS v. LPS + Low GLP-2; (3) LPS v. LPS + High GLP-2; Values are means of ten replicates

### Plasma GLP-2 concentration

To investigate the endogenous GLP-2 secretion, the basal level of plasma GLP-2 was examined. Plasma GLP-2 concentrations for the four treatment groups (pre-LPS challenge) are shown in Table 2. Plasma GLP-2 levels changed significantly after weaning. In the control and low GLP-2 groups, plasma GLP-2 was decreased significantly on d 7 (*P <* 0.001 and *P <* 0.001) and subsequently increased to the weaning level on d 14 (*P =* 0.231 and 0.055). In the pre-LPS group (injected with sterile PBS), the plasma GLP-2 level was significantly lower than the weaning level on d 7 and 14 postweaning (*P <* 0.001 and *P =* 0.006). However, in the high GLP-2 group, plasma GLP-2 was maintained on d 7 (*P =* 0.522) and then increased to a higher level on d 14 than that was on the weaning day (*P <* 0.001). Compared with the pre-LPS group, the reduction of plasma GLP-2 level on d 7 postweaning was suppressed by both GLP-2 injections (*P <* 0.001and *P <* 0.001). The plasma GLP-2 level in the low and high GLP-2-treated groups was significantly higher than that of the pre-LPS group on d 14 postweaning (*P =* 0.001 and *P <* 0.001).

**Table 2:** The effect of h[Gly2]GLP-2_1-34_ on plasma GLP-2 concentrations in weaned piglets before LPS challenge^a^ (pg/mL)

Item		Treatment^b^				Pooled SEM	Contrast^d^		
		Control	LPS	LPS + Low GLP-2	LPS + High GLP-2		1	2	3
d 0		199.8	202.1	200.7	201.6	1.7	0.646	0.773	0.921
d 7		169.4	164.2	179.9	195.2	2.2	0.120	<0.001	<0.001
d 14		195.1	190.6	210.4	230.1	3.1	0.397	0.001	<0.001
Pooled SEM		2.9	3.3	3.0	3.5	--	--	--	--
Contrast^c^	1	<0.001	<0.001	<0.001	0.522	--	--	--	
	2	<0.001	<0.001	<0.001	<0.001	--	--	--	
	3	0.231	0.006	0.055	<0.001	--	--	--	

^a^GLP-2 = Glucagon-like peptide 2; LPS = *Escherichia coli* lipopolysaccharide

^b^Control: non-challenged control; LPS: pigs injected with sterile PBS solution containing 0 nmol/kg BW human GLP-2; LPS + Low and LPS + High GLP-2: piglets injected with PBS solution containing h[Gly2]GLP-2_1-34_ at doses of 2 and 10 nmol/kg BW per day for the first 7 d after weaning. The data were obtained before LPS challenge

^c^Contrast: (1) 0 d v. 7 d; (2) 7 d v. 14 d; (3). 0 d v.14 d

^d^Contrast: (1) control v. LPS; (2) LPS v. LPS + Low GLP-2; (3) LPS v. LPS + High GLP-2; Values are means of ten replicates

### Intestinal weight and morphology

**Fig. 2:**
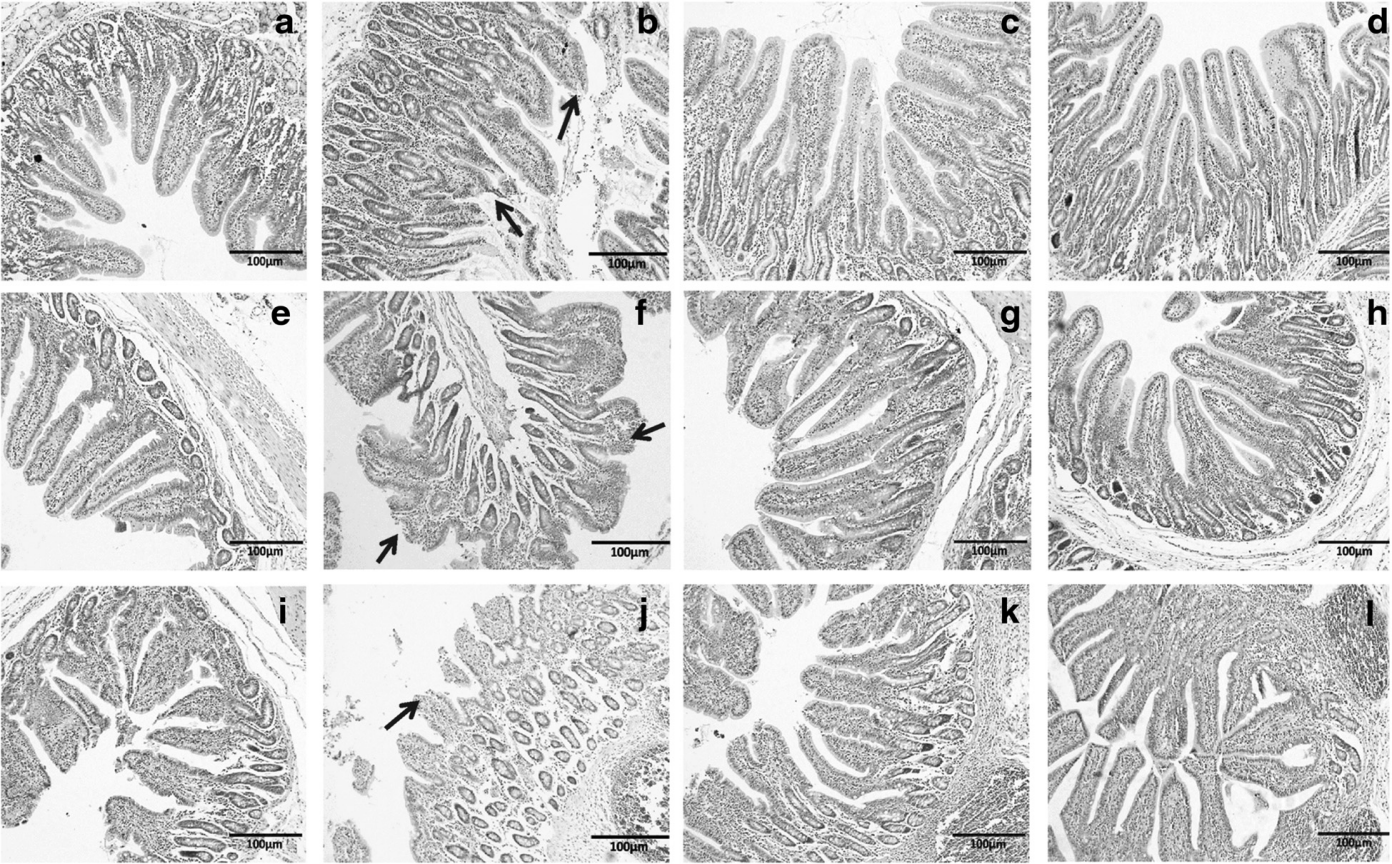
GLP-2 pretreatment maintained the integrity of the villus, and protected it against LPS-induced damage. Micrographs of duodenal (**a**, **b**, **c**, **d**), jejunal (**e**, **f**, **g**, **h**), and ileal (**i**, **j**, **k**, **l**) villi from control, LPS-treated, and GLP-2-treated piglets at 15 d postweaning. Control group (**a**, **e**, **i**); LPS group (**b**, **f**, **j**); 2 nmol/kg GLP-2 group (**c**, **g**, **k**); and 10 nmol/kg GLP-2 group (**d**, **h**, **l**). The arrows in panels **b**, **f** and **j** indicate the damaged tips of the intestinal villi after LPS treatment. Desquamation was observed at the tips of the intestinal villi, and the exposed lamina propria was clearly seen in the control group (panels **b**, **f** and **j**). (100 ×)

The effects of hGLP-2 on the weight of the duodenum, jejunum, and ileum after LPS challenge are shown in Table 3. LPS challenge significantly decreased the duodenal, jejunal and ileal weight, as well as the gross weight of SI, and the SI weight index compared with the control pigs (*P =* 0.008, 0.034, 0.035, 0.017 and 0.016). The low GLP-2 treatment significantly increased the duodenal and jejunal weight, as well as the gross weight of SI (*P =* 0.019, 0.042 and 0.026). The high GLP-2 dose significantly increased the duodenal, jejunal and ileal weight, as well as the gross weight of SI, and the SI weight index (*P =* 0.005, 0.003, 0.001, 0.001 and 0.015). The morphology of the duodenum, jejunum, and ileum after LPS challenge are shown in Table 4. Compared with control pigs, LPS pigs had lower villus height (*P <* 0.001, *P =* 0.001 and *P* < 0.001), and lower VCR (*P <* 0.001, *P =* 0.005 and *P* < 0.001) in the duodenum, jejunum and ileum. However, LPS did not affect crypt depth in the duodenum, jejunum and ileum. The low GLP-2 treatment significantly increased duodenal, jejunal and ileal villus height (*P <* 0.001, *P =* 0.019 and 0.041), and duodenal VCR (*P <* 0.001) when compared to the LPS group. The high GLP-2 treatment induced a significant increase of duodenal, jejunal and ileal villus height (*P <* 0.001, *P <* 0.001 and *P <* 0.001) and VCR (*P <* 0.001, *P =* 0.002 and *P <* 0.001) compared with LPS pigs. Figure 2 shows the micrographs of the duodenal, jejunal, and ileal villi in the control, LPS, and GLP-2 groups after LPS challenge. In general, blunted and shortened villi were observed in the LPS groups (panels B, F and J), while well-developed, finger-like, longer villi were observed in the GLP-2 groups. The LPS groups exhibited LPS-damaged villi with erosion of the surface epithelium at the apex of the villi (panels B, F and J). However, in the groups pretreated with GLP-2, the integrity of intestinal morphology was largely maintained. Glucagon-like peptide 2 supplementation therefore prevented the intestinal mucosal injury caused by LPS.

**Table 3:** The effect of h[Gly2]GLP-2_1-34_ on the weight of intestinal segment in weaned piglets post-LPS challenge^a^

Item	Treatment^b^				Pooled SEM	Contrast^c^		
	Control	LPS	LPS + Low GLP-2	LPS + High GLP-2		1	2	3
Duodenum, g	17.8	15.2	17.4	17.9	0.4	0.008	0.019	0.005
Jejunum, g	325	287	323	342	7	0.034	0.042	0.003
Ileum, g	43.8	38.5	42.2	47.1	1	0.035	0.134	0.001
Gross weight of SI, g	386	340	383	407	7	0.017	0.026	0.001
SI weight index	0.0402	0.0362	0.0385	0.0402	0.0006	0.016	0.147	0.015

^a^GLP-2 = Glucagon-like peptide 2; LPS = *Escherichia coli* lipopolysaccharide; SI = Small intestine; SI weight index = Gross weight of SI/BW

^b^Control: piglets injected with sterile saline on d 14 postweaning; LPS: pigs challenged by LPS on d 14 postweaning; LPS + Low and LPS + High GLP-2: piglets pretreated with GLP-2 at doses of 2 and 10 nmol/kg BW per day for the first 7 d after weaning were challenged by LPS on d 14 postweaning. The data were obtained 24 h after injection with LPS and 15 d post-weaning

^c^Contrast: (1) control v. LPS; (2) LPS v. LPS + Low GLP-2; (3) LPS v. LPS + High GLP-2; Values are means of ten replicates

**Table 4:** Effect of h[Gly2]GLP-2_1-34_ pretreatment on the intestinal morphology of weaned pigs exposed to LPS^a^

Item	Treatment^b^				Pooled SEM	Contrast^c^		
	Control	LPS	LPS + Low GLP-2	LPS + High GLP-2		1	2	3
Duodenum								
Villus height, μm	438	335	420	438	8	<0.001	<0.001	<0.001
Crypt depth, μm	242	257	264	237	3	0.094	0.415	0.027
VCR	1.81	1.31	1.60	1.85	0.04	<0.001	<0.001	<0.001
Jejunum								
Villus height, μm	341	284	322	353	7	0.001	0.019	<0.001
Crypt depth, μm	210	207	227	212	3	0.708	0.017	0.477
VCR	1.63	1.38	1.43	1.67	0.03	0.005	0.607	0.002
Ileum								
Villus height, μm	306	239	259	324	6	<0.001	0.041	<0.001
Crypt depth, μm	199.5	205.3	209.9	209.0	1.8	0.259	0.377	0.863
VCR	1.54	1.16	1.24	1.56	0.03	<0.001	0.237	<0.001

^a^GLP-2 = Glucagon-like peptide 2; LPS = *Escherichia coli* lipopolysaccharide; VCR = Villus height/Crypt depth ratio

^b^Control: piglets injected with sterile saline on d 14 postweaning; LPS: pigs challenged by LPS on d 14 postweaning; LPS + Low and LPS + High GLP-2: piglets pretreated with GLP-2 at doses of 2 and 10 nmol/kg BW per day for the first 7 d after weaning were challenged by LPS on d 14 postweaning. The data were obtained 24 h after injection with LPS and 15 d post-weaning

^c^Contrast: (1) control v. LPS; (2) LPS v. LPS + Low GLP-2; (3) LPS v. LPS + High GLP-2; Values are means of ten replicates

### Enzyme activity

The effect of GLP-2 injection on enzyme activity in the small intestine of LPS-challenged weaned piglets is shown in Table 5. Compared with the control group, piglets in the LPS group exhibited decreased activity of AKP, γ-GT, and pancreatic lipase in the duodenum (*P <* 0.001, *P =* 0.045 and 0.020) and jejunum (*P =* 0.047, 0.019 and 0.031). The high GLP-2 treatment significantly increased the activity of AKP, γ-GT, pancreatic amylase and lipase in the duodenum (*P <* 0.001, *P =* 0.004, *P =* 0.019 and *P <* 0.001), and AKP, γ-GT, and pancreatic lipase in the jejunum (*P =* 0.007, 0.008 and 0.004). The low GLP-2 treatment pigs had increased activity of AKP and pancreatic lipase in the duodenum (*P <* 0.001 and *P =* 0.019). No significant differences were observed in the activity of duodenal and jejunal Na^+^-K^+^-ATPase and jejunal amylase between the GLP-2 treated groups and the LPS group.

**Table 5:** Effect of h[Gly2]GLP-2_1-34_ on enzyme activity in the small intestine of LPS-challenged weaned piglets^a^

Item	Treatment^b^				Pooled SEM	Contrast^c^		
	Control	LPS	LPS + Low GLP-2	LPS + High GLP-2		1	2	3
Duodenum								
Na^+^-K^+^-ATPase, U/mg protein	541	511	536	522	18	0.573	0.636	0.828
AKP, U/mg protein	47.6	27.3	42.1	49.6	1.8	<0.001	<0.001	<0.001
γ-GT, U/g protein	1,035	872	1,015	1,117	30	0.045	0.079	0.004
Amylase, U/mg protein	74.5	67.6	74.3	76.3	1.3	0.060	0.068	0.019
Lipase, U/mg protein	17,777	15,227	17,792	19,608	435	0.020	0.019	<0.001
Jejunum								
Na^+^-K^+^-ATPase, U/mg protein	566	524	524	532	13	0.275	0.996	0.829
AKP, U/mg protein	15.4	12.4	14.4	16.5	0.5	0.047	0.178	0.007
γ-GT, U/g protein	988	818	945	1,013	26	0.019	0.075	0.008
Amylase, U/mg protein	154	144	166	161	4.7	0.467	0.103	0.215
Lipase, U/mg protein	48,436	38,616	44,289	52,209	1695	0.031	0.204	0.004

^a^GLP-2 = Glucagon-like peptide 2; LPS = *Escherichia coli* lipopolysaccharide; AK*P =* Alkaline phosphatase; γ-GT = γ-Glutamyltranspeptidase

^b^Control: piglets injected with sterile saline on d 14 postweaning; LPS: pigs challenged by LPS on d 14 postweaning; LPS + Low and LPS + High GLP-2: piglets pretreated with GLP-2 at doses of 2 and 10 nmol/kg BW per day for the first 7 d after weaning were challenged by LPS on d 14 postweaning. The data were obtained 24 h after injection with LPS and 15 d post-weaning

^c^Contrast: (1) control v. LPS; (2) LPS v. LPS + Low GLP-2; (3) LPS v. LPS + High GLP-2; Values are means of ten replicates

## Discussion

### Alteration of plasma GLP-2 concentration during the weaning period

The plasma concentration of GLP-2 in piglets increases before birth, peaks in 1-d-old suckling piglets, and decreases with weaning-related anorexia [6]. In this study, the plasma GLP-2 concentration declined abruptly after weaning, when intestinal adaptation occurred. Additionally, the decreased GLP-2 was followed by decreased digestibility and absorptivity. These findings reveal a relationship between the GLP-2 level and intestinal adaptation. Previous studies showed that supplementation of enteral nutrients or exogenous GLP-2 induced an increase in plasma concentration of GLP-2 in neonatal piglets and rats with intestinal injury or short bowel syndrome [3, 4, 12]. Similarly, in the present study, injection of a high dose of GLP-2 significantly prevented the decline of basal levels of plasma GLP-2 and increased the GLP-2 above the weaning level. Although exogenous GLP-2 was administered for only the first 7-day, GLP-2 levels remained high in the GLP-2-treated groups on d 14. Moreover, on d 15, LPS-induced mucosal injury was alleviated in both GLP-2-treated groups. GLP-2 plasma levels likely remained high because long-term injection of exogenous GLP-2 promotes not only the recovery of intestinal adaptability after weaning, but also the piglets’ capacity to secrete endogenous GLP-2. The latter effect may be attributed to the ability of GLP-2 to stimulate proliferation and differentiation of intestinal epithelial cells. However, the mechanism of action for this endogenous effect remains unclear. Therefore, current efforts to increase circulating levels of GLP-2 in order to modulate the intestinal environment are likely to involve exogenous supplementation of GLP-2.

### Effect of GLP-2 on gut weight and morphology

Weaning of piglets is associated with gross changes in small intestinal morphology and structure, including villous atrophy, decreased villus height, and increased crypt depth, resulting in decreased intestinal active absorption [13]. Many animal models have demonstrated a significant correlation between exogenously administered GLP-2 and increased growth parameters of the small and large intestines. For example, in one study, supplemental enteral nutrients and exogenous GLP-2 acted in synergy to induce dramatic mucosal growth in rats [4]. In other studies, GLP-2 infusion at various dosages increased small intestinal mucosal weight [14], DNA and protein content, and villus height in neonatal piglets [3]. In agreement with these previous reports, our current study demonstrates that high GLP-2 induced marked increases in the duodenal, jejunal and ileal weight, as well as the gross weight of the SI, and the SI weight index. However, the ileal weight showed no significant increase in the low GLP-2-treated piglets. The explanation for this may be due to the dose-dependent effect of GLP-2 [3], and the highly tissue-specific distribution of the GLP-2 receptor (GLP-2R), which is known to be predominantly localized to the duodenum and jejunal [15].

The architecture of the villus-crypt of the small intestine reflects the health of the small intestine. Similar to the report of Zhu et al. [9], the present study reports that LPS causes morphologic changes associated with intestinal mucosal injury, such as desquamation of epithelium at the tip of the villus and villous atrophy. Glucagon-like peptide 2 prevented LPS-induced effects, including desquamation of villous epithelium, and increased villous height and VCR of the duodenum and ileum. We conclude, therefore, that GLP-2 alleviates damage to the intestinal villi. This suggests that GLP-2 can promote the development of villus-crypt architecture and can enhance the barrier function of the intestinal mucosa. These experimental results are consistent with the findings of Drucker et al. [16] and are also consistent with previous reports that GLP-2 prevents small intestinal atrophy in total parenteral nutrition (TPN)-fed neonatal piglets [17]. The effects of GLP-2 in these studies may be attributed to its ability to increase small intestinal mesenteric blood flow [18, 19], stimulate intestinal cell survival and proliferation, and suppress cell apoptosis and proteolysis [20, 21].

### Effect of GLP-2 on enzyme activity

Prevention of intestinal villous atrophy can improve nutrient digestion and absorption of weaned piglets and eliminate their growth gap [2]. In the current study, we measured the activity of several duodenal and jejunal enzymes, including mucosal AKP, γ-GT, Na^+^-K^+^-ATPase, pancreatic amylase and lipase. Interestingly, lipase activity was greatly improved by GLP-2 treatment. This may be due to the ability of GLP-2 to promote the secretion of pancreatic lipase in the intestine by increasing pancreatic blood flow [22]. In addition, the activity of duodenal and jejunal AKP and γ-GT was increased significantly by GLP-2 treatment. Intestinal AKP is an enzymatic marker for intestinal mucosa, reflecting epithelial proliferation and intestinal absorbance capacity [23]. Many animal studies have demonstrated a positive effect of GLP-2 on intestinal enzymes. Glucagon-like peptide 2 increased jejunal maltase-glucoamylase and sucrase-isomaltase mRNA abundance and activity levels in parenterally-fed premature neonatal piglets [14]. Glucagon-like peptide 2 protected against TPN-induced intestinal hexose malabsorption [24] and acutely stimulated intestinal glucose utilization in TPN-fed piglets [25]. In addition, treatment of weaned piglets with polyethylene glycosylated porcine GLP-2 increased sucrase activity in the jejunum and lactase activity in the duodenum and jejunum [26]. Therefore, GLP-2 promotes digestive and absorptive intestinal functions. The specific mechanism of action of GLP-2 is not yet clear, but it is thought to be related to increases in blood flow [22], plasma GLP-2 concentration, and GLP-2R expression [27].

### Effect of GLP-2 on growth performance

Weaning stress can temporarily inhibit piglet growth. The present work shows that GLP-2 improves the growth performance of weaned piglets, as seen in the increased ADG and G:F. Previous experiments in rats have also demonstrated a similar effect of GLP-2 on growth performance. Whether administered immediately or delayed until inflammation was observed, GLP-2 treatment significantly increased animal BW [28]. Continuous infusion of GLP-2 at a dose of 240 μg/kg per day induced intestinal growth and increased weight gain in a rodent model of TPN mucosal atrophy [29]. The growth-promoting effects of GLP-2 may be largely attributed to the improvement of intestinal development by GLP-2.

Tang-Christensen et al. [30], Dalvi, Belsham [31], and Guan et al. [32] have demonstrated that GLP-2 is a specific neurotransmitter that inhibits rodent feeding behavior. GLP-2 is a potent anorexigenic peptide in the brain, and it can up-regulate mRNA levels of hypothalamic neuropeptides involved in appetite regulation in chicks [33]. Peripheral administration of GLP-2 reduced food intake in mice in the short term (Baldassano et al., 2012). However, in the current study, there was no difference in ADFI among the four treatments at d 0 to 7, 7 to 14 as well as d 0 to14 (pre-challenge). This is in accordance with previous reports indicating that peripheral administration of GLP-2 did not influence appetite and *ad libitum* food intake in humans [34]. The reason for this discrepancy may be that at physiological concentrations, circulating GLP-2 does not seem to play a significant role in appetite regulation [35].

## Conclusions

GLP-2 improves growth performance, promotes the development of intestinal morphology, and enhances the activity of digestive and absorptive enzymes in weaned piglets. Exogenous administration of GLP-2 (10 nmol/kg) promotes the secretion of endogenous GLP-2 to increase the basal level of GLP-2 and promotes the maintenance of GLP-2 levels even after exogenous supplementation has ceased. The mechanism by which GLP-2 enhances the activity of digestive and absorptive enzymes warrants further study.

## Abbreviations

GLP-2: glucagon-like peptide-2
LPS: *Escherichia coli* lipopolysaccharide
ADFI: Average daily feed intake
ADG: Average daily gain
G:F: gain:feed ratio
VCR: villus height/crypt depth ratio
SI: small intestine
GLP-2R: GLP-2 receptor
AKP: alkaline phosphatase
γ-GT: γ-glutamyltranspeptidase
